# The Anti-Inflammatory and Antioxidative Effects of Ningmitai Capsule in the Experimental Autoimmune Prostatitis Rat Model

**DOI:** 10.1155/2020/5847806

**Published:** 2020-05-28

**Authors:** Haicheng Chen, Yun Xie, Cuncan Deng, Chi Zhang, Linyan Lv, Jiahui Yao, Xiangzhou Sun, Chunhua Deng, Guihua Liu

**Affiliations:** ^1^Department of Andrology, The First Affiliated Hospital of Sun Yat-sen University, Guangzhou, China; ^2^Reproductive Medicine Center, The Sixth Affiliate Hospital of Sun Yat-sen University, Guangzhou, China

## Abstract

**Objective:**

Ningmitai (NMT) capsule has been widely prescribed for the treatment of chronic prostatitis/chronic pelvic pain syndrome (CP/CPPS), but the mechanism remains unclear. This study aims to evaluate the therapeutic effects of the NMT capsule in the experimental autoimmune prostatitis (EAP) rat models and explore its possible mechanisms.

**Methods:**

A total of fifty male Sprague Dawley rats were used in this study. Prostate extract was obtained for the induction of EAP rat models. The EAP rats were randomly divided into the model group, NMT low-dose group (0.45 g/kg/d), NMT medium-dose group (0.90 g/kg/d), and NMT high-dose group (1.80 g/kg/d), with six rats per group. Three NMT treatment groups were administered with the NMT capsule by gavage for 30 days. HE staining was used for histopathological analyses of prostate tissues. Western blotting was used to measure the expression of proinflammatory factors IL-1*β* and TNF-*α*. The MDA level was detected to reflect the level of oxidative stress. The bilateral dorsal root ganglia of T3/L1 to S4 were dissected to measure the substance *P* expression.

**Results:**

EAP rat models were successfully constructed, in which extensive infiltration of inflammatory cells was found. Treatment of NMT capsule for 30 days and the infiltration of inflammatory cells were significantly mitigated (*P* < 0.05), especially in the NMT medium-dose group and NMT high-dose group. Moreover, the expression of IL-1*β* and the level of MDA were significantly decreased (*P* < 0.05). In addition, NMT treatment could significantly alleviate substance *P* expression in dorsal root ganglia.

**Conclusion:**

Our findings demonstrate that the NMT capsule can alleviate inflammatory response and oxidative stress and reduce the production of substance *P* in EAP rats. This provides a theoretical basis for the clinical application of NMT capsule for CP/CPPS treatment.

## 1. Introduction

Chronic prostatitis/chronic pelvic pain syndrome (CP/CPPS) is defined as chronic pelvic pain not caused by other identifiable pathology, according to the classification of prostatitis developed by the National Institutes of Health (NIH) in 1994 [1]. It is a common disease in adult men and affects the quality of patients' life. In low-income and middle-income countries, the prevalence of chronic pelvic pain or prostatitis was 11% in the general adult populations [2]. Due to its complex pathology, the treatment of CP/CPPS remains challenging.

Although antibiotics and *α*-blockers have been widely used for the management of CP/CPPS [3], the efficacy of these treatments is usually unsatisfactory in clinical practice. In most cases, CP/CPPS is not associated with bacterial infection. Nonetheless, doctors usually choose antibiotics to treat CP/CPPS according to experience, so that the efficacy of antibiotics is doomed to be limited. *α*-Blockers are used to improve urinary symptoms of CP/CPPS patients. However, several RCTs research studies showed heterogeneous outcomes of monotherapy with *α*-blockers [4–7]. These results suggested that *α*-blockers may not be recommended as a first-line monotherapy [8].

Autoimmune inflammation is one of the main causes of CP/CPPS [9, 10]. A study showed that the expression of inflammatory protein IL-1*β* is higher in CP/CPPS patients when compared with controls [11]. On the other hand, oxidative stress also has been regarded as an important factor in the process of chronic prostatitis [12]. Hence, anti-inflammatory and antioxidative treatments may be the ideal strategies for CP/CPPS.

Ningmitai (NMT) capsule has been widely prescribed for the treatment of CP/CPPS in China. A meta-analysis [13] of thirty RCTs with 6,185 patients also found that the NMT capsule is effective and safe in the treatment of chronic prostatitis. However, the mechanism of NMT in the treatment of CP/CPPS remains unclear. NMT capsule is a pure Chinese medicine preparation consisted with Xian He Cao (*Herba Agrimoniae*), Touhualiao (*Herba Polygoni Capitati*), Bai Mao Gen (*Rhizoma Imperatae*), Dafengteng (*Radix Cocculi Trilobi*), Sankezhen (*Berberidis radix*), Furongye (*Folium Hibisci Mutabilis*), and Lianqiao (*Fructus Forsythiae suspensae*) [14]. Some of these components have been reported having anti-inflammatory and antioxidative effects in other diseases [15, 16]. Therefore, we hypothesized that NMT capsule could treat CP/CPPS through anti-inflammatory and antioxidative effects. In this study, we aim to evaluate the therapeutic effects of the NMT capsule and explore its possible mechanisms to alleviate CP/CPPS in the experimental autoimmune prostatitis rat model.

## 2. Materials and Methods

### 2.1. Animals

Fifty healthy male Sprague Dawley (SD) rats (12 weeks old) were used in this study, all of which were purchased from the Laboratory Animal Center of Sun Yat-sen University (Guangzhou, Guangdong Province, P.R. China). The animals were kept in the standard, pathogen-free environment on a 12-h light/12-h dark cycle with free access to laboratory chow and water. The animal procedures were approved by the Institutional Animal Care and Use Committee (IACUC) of Sun Yat-sen University.

### 2.2. Establishment of Experimental Autoimmune Prostatitis (EAP) Rat Models

EAP rat model could simulate the inflammatory characteristics of typical CP/CPPS. It is considered as a suitable model for human CP/CPPS [17]. To establish the EAP rat models, we followed a standard protocol previously described [18, 19]. In brief, twenty male SD rats were sacrificed to obtain prostate tissues. After weighted, prostate tissues were washed with cold physiological saline, and an equal weight of 0.5% Triton X-100 physiological saline solution was added. Homogenized with a glass homogenizer on an ice water bath. Homogenate was centrifuged at 12000 rpm/min for 30 min at 4°C. Then the supernatant was retained as prostate extract. The concentration of prostate extract was measured by the Pierce BCA Protein Assay Kit (Thermo 23225). Then prostate extract was diluted with physiological saline to 2 mg/mL. To prepare the injected mixture for the induction of autoimmune prostatitis, the diluted prostate extract was added with an equal volume of complete Freund's adjuvant (CFA) (Sigma-Aldrich, St. Louis) in a final concentration of 1 mg/mL. On day 0, each rat for EAP modeling was injected with 1 mL mixture. The rats received four intradermal injections in different places: right footpad, left footpad, base of the tail, and shoulders. On day 20, the above operation was repeated for the second injection. On the other hand, each rat in the normal group was injected with 1 mL mixture consisting of physiological saline plus CFA in the same way for controlling.

### 2.3. Study Design

Thirty SD rats were randomly divided into five groups with six rats per group: normal group (group N), model group (group Mo), NMT low-dose group (group L), NMT medium-dose group (group M), and NMT high-dose group (group H). NMT capsule was provided by Shanghai Xintian Pharmacy Company. Rats in group Mo and three NMT treatment groups had been induced into EAP as described above. Among NMT treatment groups, the therapeutic dose of NMT capsule in group L was 0.45 g/kg by gavage (0.10 g crude drug/mL), that in group M was 0.90 g/kg, and that in group H was 1.80 g/kg each day. Group N and group Mo were administered an equal volume of saline by gavage. All groups were given gavage once a day for 30 days. Then the body weight was measured. The rats were killed with an overdose injection of pentobarbital (>100 mg/kg), and their prostates were weighed up.

### 2.4. Prostatic Index Determination

The prostatic index [20] was calculated by the following equation:

PI = weight of prostrate (mg)/body weight (g).

### 2.5. Hematoxylin-Eosin (HE) Staining and Histopathological Analyses

Prostate tissues were deparaffinized in xylene and dehydrated in alcohol. Then, the tissues were stained in Harris hematoxylin solution for 8 min before bluing in 0.2% ammonia water or saturated lithium carbonate solution for 1 min. After rinsing in 95% alcohol, tissues were counterstained in the eosin-phloxine solution for 2 min. Finally, the tissues were mounted with a xylene-based mounting medium. Images were captured using a Leica confocal microscope. Then analyzed the histopathological features of the prostate gland. To evaluate the aggressiveness of inflammation, three fields of view were randomly selected from each slice, and the total numbers of inflammatory cells were counted.

### 2.6. Immunohistochemistry (IHC) Staining

The bilateral dorsal root ganglia of T3/L1 to S4 were dissected for IHC staining to evaluate the substance P (SP) expression. It was performed using a Dako Envision System (Dako, Santa Clara, CA, USA.) following the manufacturer's protocol. Sections were blocked using serum-free protein block buffer (Dako, Santa Clara, CA, USA) for 30 min, after which they were incubated with the antibodies of SP, SP-DE4-21 (sc-58591; dilution 1 : 100; Santa Cruz Biotechnology).

### 2.7. Western Blotting

The prostate protein levels were analyzed by Western blotting. In brief, total protein was extracted by RIPA Lysis Buffer and separated by polyacrylamide gels, and then, was transferred onto PVDF membranes. After blocked with 5% BSA solution, the membranes were incubated with primary antibodies, including GAPDH (T0004, dilution 1 : 5000; Affinity), *β*-actin (AF7018; dilution 1 : 5000; Affinity), IL-1*β* (AF7018; dilution 1 : 5000; Affinity), and TNF-*α* (ab6671; dilution 1 : 1000; Abcam). Then, the membranes were incubated with secondary antibodies for 1 hour at room temperature. Finally, immunodetection was performed using Chemi-Doc Imaging System (Bio-Rad, Hercules, CA, USA).

### 2.8. Oxidative Stress Index Detection

Superoxide dismutase (SOD) and malondialdehyde (MDA) were detected according to the corresponding assay kit. The SOD activity was detected by the Superoxide Dismutase assay kit (WST-1 method) (A001-3, NanJing JianCheng Bioengineering Institute). The MDA expression was detected by the Malondialdehyde assay kit (TBA method) (A003-1, NanJing JianCheng Bioengineering Institute). The prostate tissue was weighed, and a 10% tissue homogenate was made in a glass homogenizer for total protein quantification using Pierce BCA Protein Assay Kit (Thermo 23225).

### 2.9. Statistical Analysis

The statistical analysis was performed with SPSS for Windows (version 20.0, SPSS IBM Corp., Armonk, NY, USA). Continuous variables were expressed as the mean ± standard deviation. Multiple groups were compared using one-way analysis of variance (ANOVA). Values of *P* < 0.05 were considered to be statistically significant.

## 3. Results

### 3.1. Effects of NMT Capsule on EAP Rat Models

In our study, three doses of NMT capsule were administered to EAP rat models to investigate the effects of NMT capsule on CP/CPPS. The effects of NMT treatment were summarized in Table 1. As we can see, the body weight of rats was significantly decreased in the group Mo compared with that in the group N. Compared with the group Mo, the body weight of rats in the three treatment groups increased significantly. These results suggest that prostatitis in rats would cause the loss of body weight, and the treatment with NMT capsule is beneficial to restore the weight in rats. Additionally, as for the prostate index (PI), there was no significant difference among the five groups.

### 3.2. NMT Capsule Mitigated the Infiltration of Inflammatory Cells in Prostate Tissues

**Figure 1:**
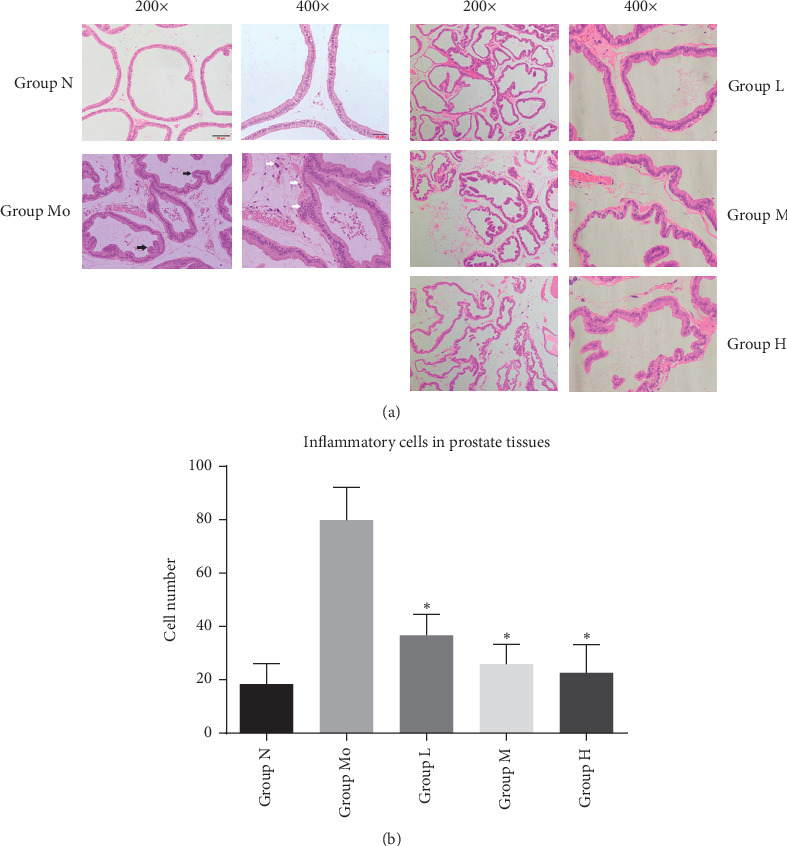
Histopathological analysis of prostate tissues. Notes: hematoxylin-eosin staining prostate tissues after treatment for 30 days. (a) The shape of the glandular cavity and luminal secretions of prostate tissues (the black arrows show the irregular glandular cavity; the white arrows show the inflammatory cells). (b) Number of inflammatory cells in prostate tissues (^*∗*^*P* < 0.05).

It was suggested in the Introduction that NMT capsule may treat CP/CPPS through the anti-inflammatory effect. To investigate the histopathological change in prostate tissues after the treatment, HE staining was used. As shown in Figure 1, histopathological features of the prostate gland were normal in the group N. The shape of the glandular cavity of the prostate was regular, and the luminal secretion was uniform. Meanwhile, there was no obvious inflammatory change in the epithelium and interstitium. In contrast, in the group Mo, the prostatic glandular cavity was irregular, and the luminal secretion was not uniform. Besides, numerous inflammatory cells were found in the interstitium. Interestingly, the inflammatory cells were reduced in NMT treatment groups. In particular, in the group M and group H, that inflammatory cells infiltration almost mitigated to normal. These results indicated that NMT capsule could mitigate the infiltration of inflammatory cells in prostate tissues. Whereas the morphology of the glandular cavity of the prostate gland was still irregular, and the endocrine secretion in the glandular cavity was uneven.

### 3.3. Effects of NMT Capsule on IL-1*β* and TNF-*α* Levels in Prostate Tissues

**Figure 2:**
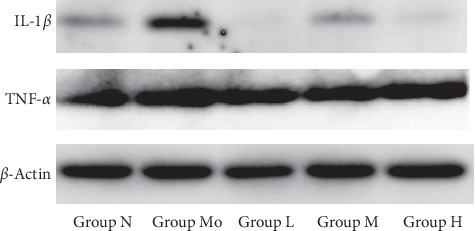
Effects of NMT capsule on IL-1*β* and TNF-*α* levels in prostate tissues.

Inflammatory factors are important in CP/CPPS development, such as IL-1*β* and TNF-*α* [21]. As expected, the expression of IL-1*β* in the model group was significantly increased in comparison with the normal group. With the treatment of NMT capsule for 30 days, it has a significant reduction of IL-1*β* expression compared with the group Mo. However, although the oral administration dose of NMT varied from 0.45 to 1.80 g/kg, there is no significant difference between the treatment groups. But the expression of TNF-*α* was not significantly different between these five groups (Figure 2).

### 3.4. NMT Capsule Reduced the Oxidative Stress Level in Prostate Tissues

**Figure 3:**
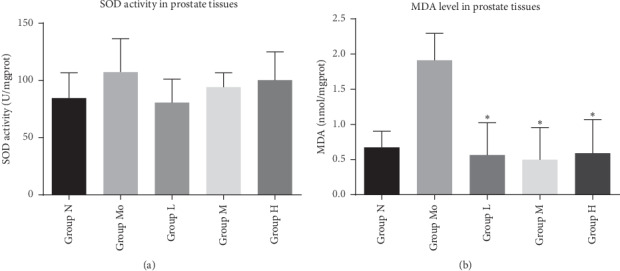
Oxidative stress level in prostate tissues. Note: oxidative stress level in prostate tissues was measured after treatment for 30 days. (a) The vitality of SOD was not significantly different between five groups; (b) the level of MDA was significantly higher in the model group. After NMT treatment, the MDA level was significantly down-regulated (^*∗*^*P* < 0.05).

The previous study also shows that oxidative stress has a significant correlation with CP/CPPS. The SOD activity and MDA level were most commonly used to evaluate the oxidative stress state. In our study, the vitality of SOD was not significantly different between the normal group, model group, and treatment group. Whereas the level of MDA was significantly higher in the model group (Figure 3, *P* < 0.05). After NMT treatment, the MDA level was significantly down-regulated (*P* < 0.05).

### 3.5. NMT Capsule Alleviated SP Expression in the Dorsal Root Ganglia

**Figure 4:**
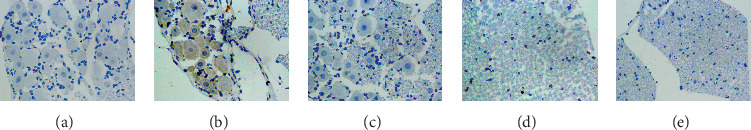
Substance *P* expression in the dorsal root ganglia. Note: substance P expression was measured after treatment for 30 days (×400). (a) Group N. (b) Group Mo. (c) Group L. (d) Group M. (e) Group H.

Pain in the pelvic, suprapubic perineal, and scrotal areas is one of the characteristics of CP/CPPS [1]. Study shows that SP released from the dorsal root ganglia have an important role in the persistent pain. [22] Hence, we detected the change of SP expression in the dorsal root ganglia. IHC staining showed that the expression of SP was not detected in the dorsal root ganglia of normal rats. Then, we found that significant SP expression can be detected in the dorsal root ganglia of the EAP rat model. Importantly, the treatment of NMT capsule could significantly alleviate the SP expression (Figure 4).

## 4. Discussion

Clinically, the NMT capsule is often used to treat CP/CPPS [13]. After treatment, patients' pelvic pain, lower urinary tract symptoms, and other discomforts can be alleviated effectively. In this study, we successfully established the EAP rat model to investigate the mechanism of the NMT capsule for CP/CPPS treatment. Based on this model, we found that the NMT capsule could treat chronic prostatitis by mitigating the infiltration of inflammatory cells and reducing IL-1*β* and MDA levels in the prostate tissues. These results suggested that NMT capsule is protected against prostatitis through anti-inflammatory and antioxidative effects.

Previous studies suggest that autoimmune inflammation is one of the main causes of CP/CPPS [9, 10, 23, 24]. Numerous proinflammatory cytokines such as IL-1*β*, TNF-*α*, IFN-*γ*, IL-6, and IL-8 are elevated in CP/CPPS [21, 24–26]. These proinflammatory factors can induce the differentiation of CD4^+^ cells, the most common immune cells in chronic prostatitis, and mediate inflammatory responses and pain. Among them, IL-1*β* exhibits a strong proinflammatory characteristic and is involved in certain autoimmune diseases [27]. Studies have shown that familial cold autoinflammatory syndrome and Muckle–Wells syndrome, which are characterized by the excessive inflammatory response in various organs, are caused by increased IL-1*β* secretion [28, 29]. Therefore, reducing the level of IL-1*β* secretion is an effective treatment for such autoimmune diseases. In this study, we similarly found that IL-1*β* increased significantly in the EAP model group. With the treatment of NMT capsule, the level of IL-1*β* can be restored to the normal level. It indicated that NMT capsule can effectively control the excessive inflammatory response in prostate tissue and effectively treat CP/CPPS by reducing the secretion of IL-1*β*.

To date, oxidative stress has been regarded as an important factor in the process of chronic prostatitis [12, 30]. In tissues of chronic prostatitis, infiltration of inflammatory cells and accumulation of inflammatory factors promote the release of cyclooxygenase-2(COX-2), transcription factor nuclear factor-kappa B (NF-*κ*B), and inducible nitric oxide synthase (iNOS) [31]. These substances further increase the formation of peroxynitrite and other toxic metabolites. In chronic prostatitis, the accumulation of peroxides can cause damage to the prostate and other organs. Isoprostane 8-epi prostaglandin-f2 (PGF2), one of the metabolic products of oxidative stress, can stimulate smooth muscle contraction in the bladder and cause urinary frequency, urgency, and other symptoms of chronic prostatitis [32]. MDA, an important product of lipid peroxidation, is used as a biomarker of oxidative stress [33, 34]. We found that the MDA level was significantly increased in the model group and significantly decreased after NMT treatment, suggesting that the NMT capsule could treat CP/CPPS through antioxidant effects.

Currently, the pelvic and suprapubic perineal pain associated with CP/CPPS is believed to be caused by dorsal root ganglia axon reflex, in which the release of SP mediates this persistent pain status [22]. Chemical irritation of the prostate resulted in significant expression of c-fos positive cells and SP in the lumbosacral spinal cord [35, 36]. Also, SP is considered a major mediator of neurogenic inflammation. In tissues, SP could stimulate mast cells to release histamine, serotonin, cytokines, chemokines, prostaglandins, and neuropeptides, which induces vasodilatation, plasma extravasation, and hypersensitivity [37]. In this study, we also found that the expression of SP was upregulated in the dorsal root ganglia of the EAP rat model and could be well downregulated with NMT treatment. It revealed why the NMT capsule can effectively relieve chronic pelvic pain of CP/CPPS patients.

NMT capsule is a pure Chinese medicine preparation consisted with Xian He Cao (*Herba Agrimoniae*), Touhualiao (*Herba Polygoni Capitati*), Bai Mao Gen (*Rhizoma Imperatae*), Dafengteng (*Radix Cocculi Trilobi*), Sankezhen (*Berberidis radix*), Furongye (*Folium Hibisci Mutabilis*), and Lianqiao (*Fructus Forsythiae suspensae*) [14]. Recent pharmacological research indicated that some components of the NMT capsule have anti-inflammatory effects. A previous study [15] showed that Xian He Cao (*Herba Agrimoniae*) could suppress lipopolysaccharide-induced nitric oxide production in BV2 microglial cells. Furthermore, it also suppressed the lipopolysaccharide-induced production of IL-1*β*, TNF-*α*, and IL-6 in a dose-dependent manner. Liao et al. [16] found that Touhualiao (*Herba Polygoni Capitati*) contains substances of certain structural types and with antibacterial and anti-inflammatory activities. These studies suggest that these compounds may play an anti-inflammatory and antioxidative role in the treatment of CP/CPPS. In this study, we failed to identify which component of the NMT capsule playing the major role, since the NMT capsule is traditional Chinese medicine preparation containing a variety of complex ingredients. Furthermore, investigations are needed to explore which component plays a key role.

In summary, our findings demonstrate that the NMT capsule can alleviate inflammatory response and oxidative stress and reduce the production of substance P in an autoimmune prostatitis rat model. This provides a theoretical basis for the clinical application of NMT capsule for CP/CPPS treatment.

## Figures and Tables

**Table 1:** Effects of NMT capsule treatment on the prostate weight, body weight, and PI in the experimental autoimmune prostatitis rat model.

Groups	Prostatic weight, PW (mg)	Body weight, BW (g)	PI, PW/BW (mg/g)
Group N	1056.33 ± 190.42	520.67 ± 17.96	2.03 ± 0.36
Group Mo	872.33 ± 161.95	508.78 ± 61.04^a^	1.72 ± 0.28
Group L	957.00 ± 209.82	542.50 ± 29.04^b^	1.76 ± 0.37
Group M	924.17 ± 154.87	605.33 ± 62.47^b^	1.55 ± 0.35
Group H	905.83 ± 144.07	583.17 ± 76.61^b^	1.56 ± 0.26

Values are mean ± SD of six animals; a: compare to the group N, *P* < 0.05; b: compare to the group Mo, *P* < 0.05.

## Data Availability

The data used to support the findings of this study are included within the article.
